# Identification of SSBP1 as a ferroptosis-related biomarker of glioblastoma based on a novel mitochondria-related gene risk model and in vitro experiments

**DOI:** 10.1186/s12967-022-03657-4

**Published:** 2022-09-30

**Authors:** Jun Su, Yue Li, Qing Liu, Gang Peng, Chaoying Qin, Yang Li

**Affiliations:** 1 Department of Neurosurgery, Hunan Children’s Hospital, No. 86 Ziyuan Road, Changsha, 410007 Hunan China; 2 Department of Neurosurgery, Xiangya Hospital, Central South University, 87 Xiangya Road, Changsha, 410008 Hunan China

**Keywords:** Glioblastoma, Mitochondria, SSBP1, Temozolomide, Ferroptosis

## Abstract

**Background:**

Glioblastoma (GBM) is the most common primary malignant brain tumor that leads to lethality. Several studies have demonstrated that mitochondria play an important role in GBM and that mitochondria-related genes (MRGs) are potential therapeutic targets. However, the role of MRGs in GBM remains unclear.

**Methods:**

Differential expression and univariate Cox regression analyses were combined to screen for prognostic differentially-expressed (DE)-MRGs in GBM. Based on LASSO Cox analysis, 12 DE-MRGs were selected to construct a risk score model. Survival, time dependent ROC, and stratified analyses were performed to evaluate the performance of this risk model. Mutation and functional enrichment analyses were performed to determine the potential mechanism of the risk score. Immune cell infiltration analysis was used to determine the association between the risk score and immune cell infiltration levels. CCK-8 and transwell assays were performed to evaluate cell proliferation and migration, respectively. Mitochondrial reactive oxygen species (ROS) levels and morphology were measured using a confocal laser scanning microscope. Genes and proteins expression levels were investigated by quantitative PCR and western blotting, respectively.

**Results:**

We identified 21 prognostic DE-MRGs, of which 12 DE-MRGs were selected to construct a prognostic risk score model for GBM. This model presented excellent performance in predicting the prognosis of patients with GBM and acted as an independent predictive factor. Functional enrichment analysis revealed that the risk score was enriched in the inflammatory response, extracellular matrix, and pro-cancer-related and immune related pathways. Additionally, the risk score was significantly associated with gene mutations and immune cell infiltration in GBM. Single-stranded DNA-binding protein 1 (SSBP1) was considerably upregulated in GBM and associated with poor prognosis. Furthermore, *SSBP1* knockdown inhibited GBM cell progression and migration. Mechanistically, *SSBP1* knockdown resulted in mitochondrial dysfunction and increased ROS levels, which, in turn, increased temozolomide (TMZ) sensitivity in GBM cells by enhancing ferroptosis.

**Conclusion:**

Our 12 DE-MRGs-based prognostic model can predict the GBM patients prognosis and 12 MRGs are potential targets for the treatment of GBM. SSBP1 was significantly upregulated in GBM and protected U87 cells from TMZ-induced ferroptosis, which could serve as a prognostic and therapeutic target/biomarker for GBM.

**Supplementary Information:**

The online version contains supplementary material available at 10.1186/s12967-022-03657-4.

## Background

Glioblastoma (GBM) is the most common and lethal malignant tumor of the central nervous system. Owing to its highly invasive nature and lack of effective therapeutic methods, its prognosis remains poor. The median overall survival (OS) of patients with GBM is < 2 years [1]. Currently, maximum tumor resection combined with radiochemotherapy remains the standard treatment for GBM. Recently, genome-wide molecular profiling studies have identified many target genes that have advanced our understanding of GBM tumorigenesis and chemoresistance. Based on these studies, several individualised therapies and novel therapeutic strategies have been developed. For example, patients with O6-methylguanine-DNA-methyltransferase (MGMT) promoter methylation may have a more effective treatment response and better prognosis [2]. However, none of these individualised targeted therapies have been shown to improve patient prognosis owing to the considerable heterogeneity between different glioma subtypes. Therefore, there is an urgent need to identify novel molecular targets and develop effective therapies for GBM.

Mitochondria perform multifaceted roles in normal physiology, including energy conversion, apoptosis regulation, biosynthetic metabolism, and cellular proliferation [3, 4]. They are vital for stress sensing, environmental adaptation, and tumorigenesis [5]. They are also involved in tumor development, progression, and treatment resistance by overproducing reactive oxygen species (ROS), which induce genomic instability, and regulate gene expression and signaling pathways [6–10]. An increasing number of studies have demonstrated that mitochondria play an important role in gliomas, including GBM. Mitochondrial DNA (mtDNA) alterations are associated with cellular and metabolic consequences, diagnosis, prognosis, and treatment of GBM [11]. Mitochondrial dynamics are reportedly essential for the development of gliomas. Dynamin-related protein 1 (*DRP1*), a key mediator of mitochondrial fission, upregulation is correlated with poor prognosis in GBM, and *DRP1* knockdown decreases glioma cell proliferation, migration, and invasiveness [12, 13]. In addition, mitochondria related ROS are involved in the oncogenesis of gliomas at various phases, such as tumor initiation and progression [14]. Excessive ROS production induced by mitochondrial damage simulataneously activates autophagy and apoptosis in GBM cells [15]. According to recent studies, anticancer agents directly targeting mitochondria related genes bypass drug resistance and improve the prognosis of patients with GBM [16, 17]. Therefore, a comprehensively analysis of mitochondria-related genes (MRGs) and exploration of their function in GBM might be useful for identifying novel prognostic biomarkers and developing effective therapeutic strategies for GBM.

In this study, we identified prognostic MRGs and constructed and validated a novel prognostic model for GBM base on 12 differentially-expressed (DE)-MRGs. Our model presented remarkable performance in predicting the prognosis of GBM patients and was confirmed to be an independent risk predictive factor. Furthermore, our risk score was enriched in inflammatory response, extracellular matrix, and pro-cancer-related and immune related pathways and closely associated with gene mutation and immune cell infiltration. To confirm the importance of these 12 DE-MRGs, we selected single-stranded DNA-binding protein 1 (SSBP1) for further in vitro studies and found that it was upregulated in GBM tissues. Furthermore, we demonstrated that *SSBP1* knockdown significantly inhibits GBM cells proliferation and migration by disturbing mitochondrial function. *SSBP1* knockdown also enhances temozolomide (TMZ) sensitivity by enhancing ROS induced ferroptosis.

## Materials and methods

### Data acquisition

The gene expression profiles of The Cancer Genome Atlas (TCGA)-GBM cohort (count and tpm) and clinical information were downloaded from the GDC Data Portal (https://portal.gdc.cancer.gov/). In addition, the single cell data of GSE84465 and the external independent GBM databases, GSE147352 and GSE16011, were gained from the Gene Expression Omnibus (GEO, https://www.ncbi.nlm.nih.gov/gds). The samples with complete survival information were retained for analysis. The gene expression data of Chinese Glioma Genome Atlas (CGGA) GBM cohort were downloaded from http://www.cgga.org.cn/.

### Identification of prognostic mitochondrial-related genes (MRGs) in GBM

First, we analysed the single-cell data of GSE84465 to identify differentially expressed genes (DEGs) between neoplastic and non-neoplastic cells by using “Seurat” package. The threshold for data filtering included minimum cells = 3, 200 < nFeature RNA < 7500, and percentage of ribosome RNA < 15. FindMarkers function was used to DEGs. based on the following criterions: logFC > 0.25 and adjusted p value < 0.05. The 686 MRGs (Additional file 6: Table S1) were obtained from the uniport database (https://www.uniprot.org/). By taking the intersection of DEGs and MRGs, we finally got 201 differentially-expressed (DE)-MRGs, of which 197 DE-MRGs can be matched in the TCGA GBM database. Subsequently, univariate Cox analysis was performed to identify the prognostic DE-MRGs based on the TCGA GBM cohort. The FeaturePlot function was used to present gene expression on a dimensional reduction plot between cell clusters.

### Construction and validation of prognostic risk score model based on the DE-MRGs

Based on the TCGA cohort, the Least absolute shrinkage and selection operator (LASSO) Cox regression analysis was used for further dimensionality reduction of prognostic DE-MRGs. Finally, 12 DE-MRGs were selected to the construct a prognostic risk model. The formula for risk score was as follows: Risk score = 0.2132 * expression level of PLAUR + 0.0261 * expression level of RBP1 + 0.0048 * expression level of ABCB8 + 0.2553 * expression level of TOMM7 − 0.1868 * expression level of MFF + 0.0714 * expression level of SSBP1 + 0.1927 * expression level of MRPL36 + 0.1686 * expression level of AGK + 0.07 * expression level of HK1—0.1339 * expression level of APEX1 + 0.2896 * expression level of NUDT1 − 0.3503 * expression level of PHB2. The Kaplan–Meier (K-M) analysis, univariate cox and multivariate cox analysis, and time dependent Receiver Operation Characteristic (ROC) curve were used to reveal the prognostic value of our model. The GSE16011 and GSE147352 GBM cohorts were used to validate this prognostic model.

### Gene Oncology (GO) and pathway enrichment analysis of our risk model

Firstly, The R package (Deseq2) was used to identify the DEGs between high- and low-risk groups based on the TCGA GBM cohort. The standards for DEGs were logFC > 1 and adjusted p value < 0.05. Then, the GO analysis of biological process (BP), cellular component (CC), and molecular function (MF) was performed by using DAVID (https://david.ncifcrf.gov/) based on the DEGs between risk groups. The pathway enrichment analysis of KEGG and HALLMARK gene sets was performed via GSAE (gene set enrichment analysis) method. An adjusted p-value < 0.05, q-value < 0.05, and absolute normalised enrichment score (NES) > 1 were used as the threshold for determination of significance.

### Ferroptosis score

Ferroptosis-related gene sets, including driver and suppressor genes, were obtained from FerrDb (http://www.zhounan.org/ferrdb/current/), the first database dedicated to ferroptosis regulators and ferroptosis-disease associations. The ferroptosis score of each sample was calculated using the single-sample GSEA (ssGSEA) method.

### Immune infiltration analysis

The infiltration levels of 28 immune cells in each sample were assessed by using single sampleGSEA based on the R package (GSVA). Additionally, the ESTIMATE algorithm was also used for calculated the ImmuneScore, StromalScore, and ESTIMATEScore of each sample based on the “estimate” package in R.

### Cell culture and transfection

All glioma cell lines (U87, U251, and SHG-44) were obtained from the Shanghai Life Academy of Sciences Cell Library (Shanghai, China). Glial cells (HEB) were obtained from the Sun Yat-Sen University Cancer Center. U87 cells were grown at 37 °C and 5% CO_2_ in Dulbecco’s modified Eagle’s medium (DMEM; HyClone, United States) supplemented with 10% foetal bovine serum. TMZ was obtained from Sigma-Aldrich Corporation.

All small interference RNA (siRNA) against the target genes and negative control siRNA were synthesised by GenPharma (Suzhou, China). U87 cells were transfected using Lipofectamine® RNAiMAX Transfection Reagent (Invitrogen, Carlsbad, California, United States), according to the manufacturer’s instructions. The sequences of the SSBP1 siRNA were as follows: siRNA #1 F 5’-CAACAAUCAUAGCUGAUAAUA, 3’-UUAUCAGCUAUGAUUGUUGUU; siRNA #2 F 5’- UAAUACAGGUCUUCGAAACAU, 3’-GUUUCGAAGACCUGUAUUACA.

### RNA extraction and quantitative PCR (qPCR)

Total RNA was extracted from U87 cells using TRIzol reagent (Invitrogen, Carlsbad, CA, United States) and reverse transcription was performed using PrimeScript™ RT Reagent Kit (Takara, Dalian, China). Real-time PCR was performed using SYBR Green Real-Time PCR Kit (Takara, Dalian, China). β-Actin was used as a normalising control. The relative expression levels were evaluated using the 2-ΔΔCT method. The primer sequences used in this study are included in Additional file 7: Table S2.

### Cell proliferation and migration assay

U87 cells were seeded in 96-well plates at a density of 5000 cells per well, Cell viability was measured using the Cell Counting Kit-8(Sigma-Aldrich, Shanghai, China) according to the manufacturer’s instructions. After knocking down SSBP1 for 72 h, U87 cells were seeded into the upper chambers at a density of 5.0 × 10^4^ cells in 300 µl of serum-free cell culture medium, while 500 µl of medium containing 20% FBS was added into the lower chambers. The cell migration assay was performed using 24-well transwell chambers (Corning, NY, United States) according to the manufacturer’s instructions.

### Measurement of ROS level, iron content, and GSH level

Mitochondrial ROS and mitochondrial membrane potential (MMP) were detected using MitoSOX™ Red Mitochondrial Superoxide Indicator (Invitrogen, United States) and MitoTracker™ Red CMXRos (Invitrogen, United States), respectively. For 24 h, 1.0 × 10^5^ U87 cells were cultured in a Nunc™ Glass Bottom Dish (Invitrogen, United States). Cells were washed with warm DPBS before incubation with 2.5 µM MitoSOX™ Red or 100 nM MitoTracker™ Red. Fluorescence intensity was analysed using a Zeiss LSM 800 confocal microscope and measured using ImageJ software.

U87 cells were transfected with siRNA and incubated with and without 400 μM TMZ for 48 h and collected. Iron content (Abcam, Cambridge, UK) and intracellular GSH levels (Abcam, Cambridge, UK) were measured according to the manufacturer’s instructions.

### Western blotting

Glioma cells and HEB were lysed in 300 µL SDS sample buffer (Sangon Biotech, China) containing 1 mM phosphatase inhibitor and 1 mM PMSF, and denatured proteins (20 µg) were resolved on 15% SDS PAGE gels and transferred to PVDF membranes. After blocking with 5% milk at room temperature, the membranes were incubated with the primary antibody overnight at 4 °C, followed by incubation with a horseradish peroxidase–conjugated secondary antibody for 2 h at room temperature. After washing the membranes thrice with TBST, ECL Reagent (Sangon Biotech, China) was added for chemiluminescent detection.

### Immunofluorescence

U87 cells were seeded in a six-well plate with three coverslips per well and cultured for 72 h at 37 °C. Further, 100 nM MitoTracker™ Red CMXRos (Invitrogen, United States) were incubated with U87 cells at 37 °C for 30 min before fixing in 4% paraformaldehyde for 10 min at room temperature. Permeabilization was performed using 0.5% Triton X-100 for 10 min at room temperature. The slides were washed and blocked with 1% BSA for 1 h. The primary antibodies were then incubated with U87 cells overnight at 4 °C. Next morning, the slides were incubated with Alexa Fluor 488 (Abcam, Shanghai, China) for 1 h at room temperature. Cell nuclei were stained with DAPI (Boyetime, Wuhan, China). Immunofluorescence images were observed using a Zeiss LSM 800 confocal microscope and analysed using ImageJ software.

### Statistical analysis

All results were analysed using GraphPad Prism 9 and R software with R packages. The experimental data were presented as means ± SDs (standard deviations) and unpaired Student’s t-test was used for continuous variables between groups. The log-rank test was utilized for K-M survival analysis. The Pearson’s correlation coefficient was applied to calculate the correlation between the expression of mtDNA-encoded genes and SSBP1. A two-side p < 0.05 was considered to indicate a statistically significant result.

## Results

### Identification of prognostic DE-MRGs in GBM

**Fig. 1:**
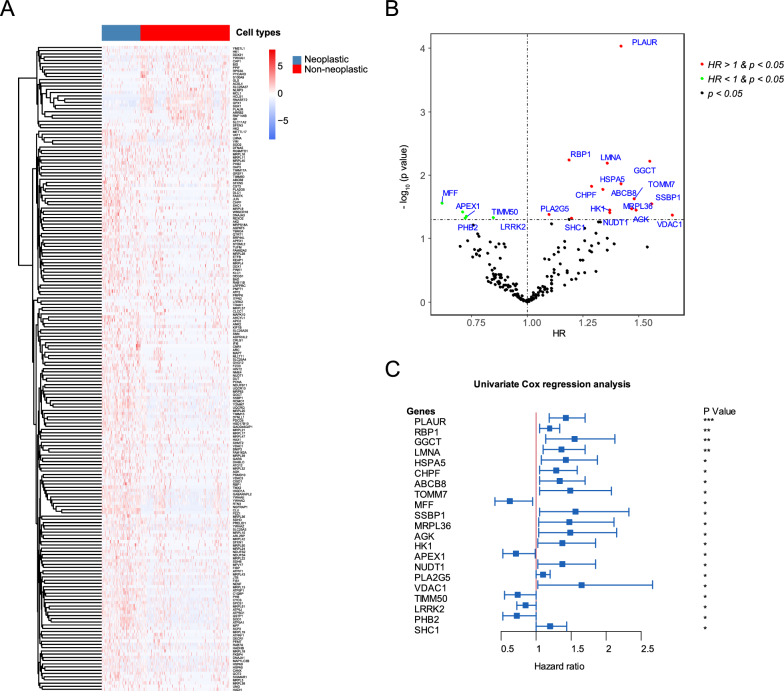
Identification of prognostic DE-MRGs in GBM. **A** The heatmap plot illustrated the 201 significant DE-MRGs between primary neoplastic and non-neoplastic cells based on the single cell data of GBM. **B** The volcano plots showed the results of univariate cox analysis of DE-MRGs in the TCGA GBM dataset. **C** The forest plot showed the 21 prognostic DE-MRGs in the TCGA GBM dataset

Differential expression analysis of the GBM single-cell dataset revealed that 201MRGs, including 172 upregulated and 29 downregulated genes, were significantly differentially expressed between neoplastic and non-neoplastic cells (Fig. 1A, Additional file 8: Table S3). Furthermore, to investigate the prognostic value of DE-MRGs in GBM patients, univariate Cox analysis was performed based on TCGA GBM dataset. The result revealed that among these DE-MRGs (14 genes could not match in TCGA GBM cohort), 21 DE-MRGs, including 5 genes with hazard ratio (HR) < 1 and 16 genes with HR > 1, were significantly associated with the OS of patients with GBM in TCGA dataset (Fig. 1B, C, Additional file 9: Table S4).

### Construction and validation of prognostic risk score model for GBM based on MRGs

**Fig. 2:**
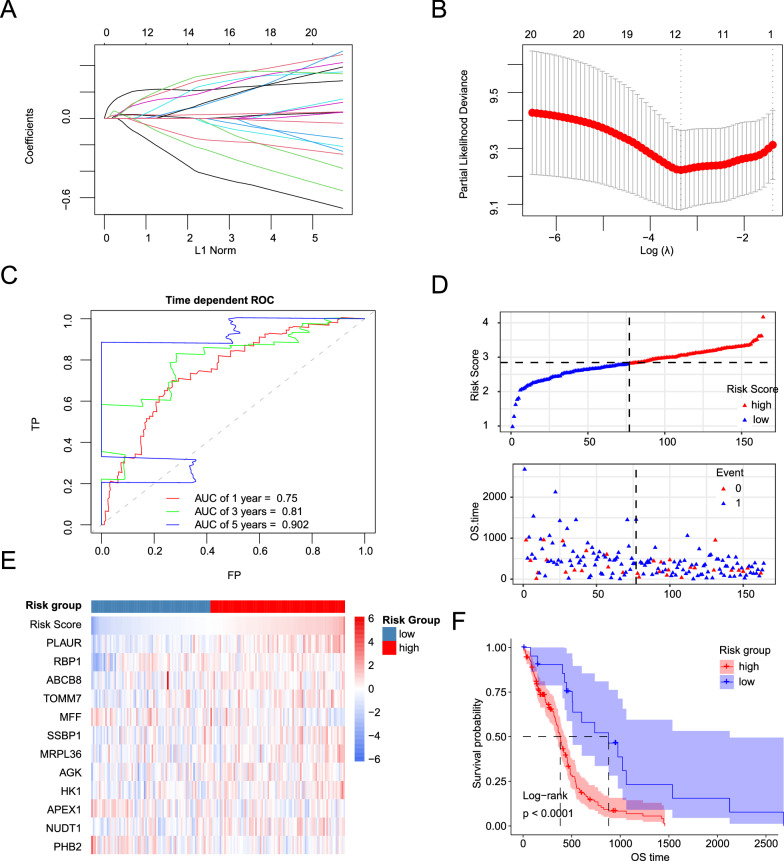
Construction of DE-MRGs related prognostic risk model for GBM. **A** The LASSO coefficient profiles of 21 prognostic DE-MRGs. **B** Ten-time cross-validation for tuning parameter selection in the LASSO model and the dotted-line equal to lambda.min. **C** Time dependent ROC curves for 12 DE-MRGS prognostic model in the TCGA GBM cohort. **D** The distribution of risk scores, survival time, and status of GBM patients in the TCGA cohort. **E** The heatmap of the 12 model DE-MRGs in the TCGA GBM cohort. **F** Kaplan–Meier curves for OS in TCGA GBM cohort stratified by 12 DE-MRGs model in high and low-risk

To further screen for potential and critical prognostic DE-MRGs, LASSO penalized Cox regression analysis was conducted. First, the coefficient values at the different penalty levels were evaluated (Fig. 2A). Next, the optimal lambda value was confirmed using a ten-fold cross-validation method (Fig. 2B). Finally,12 prognostic DE-MRGs (*PLAUR, RBP1, ABCB8, TOMM7, MFF, SSBP1, MRPL36, AGK, HK1, APEX1, NUDT1* and *PHB2*) were selected to construct a prognostic risk score model. The risk score of each GBM patient was calculated according to the formula. To evaluate the predictive accuracy and sensitivity of this prognostic model for OS, a time-dependent ROC curve analysis was performed. In TCGA GBM cohort, the areas under the ROC curve of this model for 1-, 3- and 5-year of OS were 0.75, 0.81, and 0.902, respectively, indicating that the signature of the 12 MRGs showed excellent prognostic validity (Fig. 2C). Based on the optimal cutoff value (2.33391033) for 3-year OS, patients with GBM were divided into high- and low-risk groups. The distribution of risk scores and survival status, between the high-risk group and low-risk group in TCGA-GBM cohort is presented in Fig. 2D. Heatmap showed that nine genes were highly expressed and three genes were expressed at low levels in the high-risk group (Fig. 2E). The Kaplan–Meier (KM) survival analysis curves showed that a low-risk score was related to a better prognosis in TCGA cohorts (Fig. 2F). Importantly, similar results were obtained in the GSE147352 and GSE16011 datasets, indicating that our risk model presents excellent performance in predicting the prognosis of GBM (Additional file 1: Fig. S1). We also found that our risk model was associated with the prognosis of patients with other types of tumors, such as lower grade gliomaa, lung adenocarcinoma, and kidney renal clear cell carcinoma. (Additional file 2: Fig. S2). In addition, the efficiency of our risk predictor was better than that of other similar predictors, including immune-related gene signature [18], pyroptosis-related gene signature [19], and autophagy-related gene signature [20], as reported in the literature (Additional file 3: Fig. S3).

### Prognostic model is an independent predictor and a valuable hierarchical factor

**Fig. 3:**
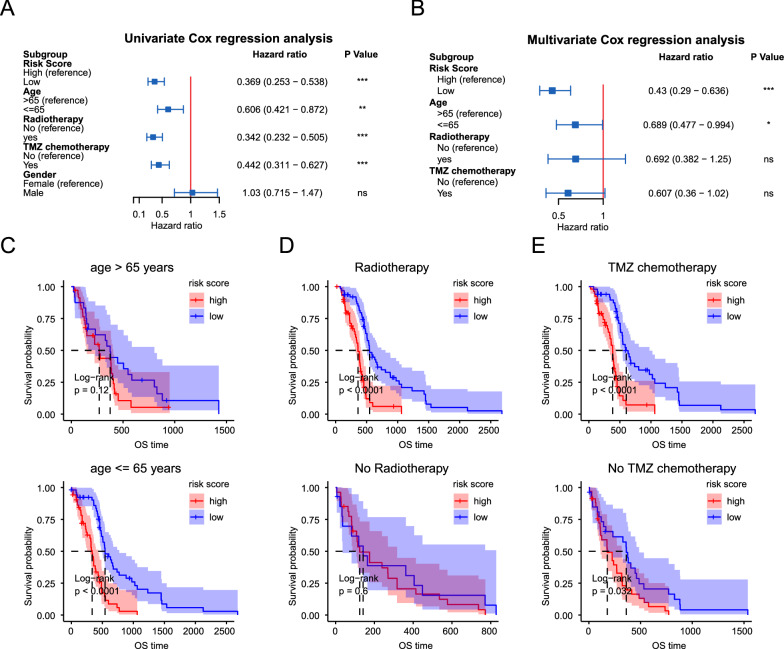
Independence of our risk score as a prognostic factor. **A** The forest plot showed the univariate cox analysis using risk score, age, radiotherapy, TMZ chemotherapy, and gender as variates in the TCGA GBM cohort. **B** The forest plot showed the multivariate cox analysis using risk score, age, radiotherapy, and TMZ chemotherapy as variates in the TCGA GBM cohort. **C** Stratified OS analysis in TCGA GBM patients with different age based on our risk model. **D** Stratified OS analysis in TCGA GBM patients with radiotherapy or not based on our risk model. **E** Stratified OS analysis in TCGA GBM patients with TMZ chemotherapy or not based on our risk model

It is well known that the age of patients and whether radiotherapy and chemotherapy affect the prognosis of patients with GBM. To assess whether our risk model is an independent predictor of the prognosis of patients with GBM, univariate and multivariate Cox analyses were performed. Univariate Cox analysis revealed that the risk score, age, and whether to receive radiotherapy or TMZ chemotherapy were significantly associated with the OS of patients (Fig. 3A). Multivariate Cox analysis demonstrated that the risk score was an independent factor for predicting OS of patients after adjusting for the abovementioned factors (Fig. 3B). Furthermore, to evaluate the prognostic values of this risk score in different subgroups of GBM, a stratified survival analysis was performed. The results showed that patients aged ≤ 65 years, received radiotherapy in the to low-risk group had better OS those in the high-risk group (Fig. 3C and D). When both TMZ chemotherapy and risk score were considered, regardless of whether they received TMZ treatment, the patients in the low-risk group had longer OS than those in the high-risk group (Fig. 3E). These results were well validated in the GSE16011 GBM cohort (Additional file 4: Fig. S4).

### Clinical and mutational characteristics of risk score

**Fig. 4:**
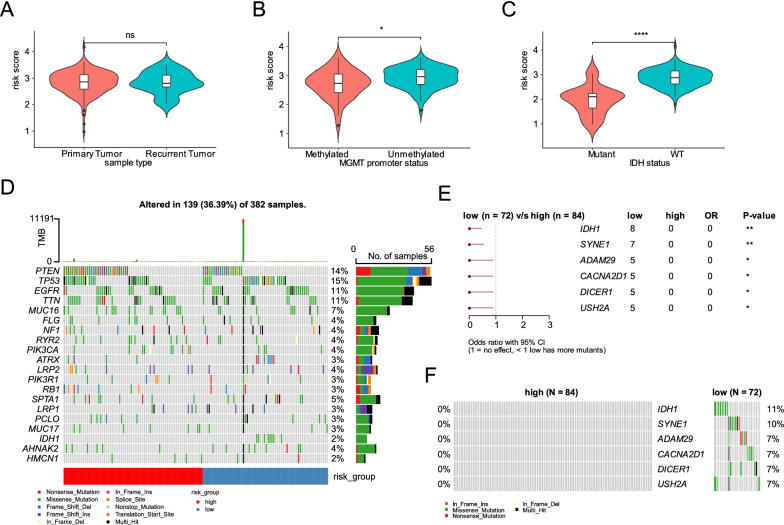
Clinical and mutational characteristics of our risk score. **A** The violin plot showed the risk score of different sample types. **B** The violin plot showed the difference of risk score between MGMT promoter methylated tumor and MGMT promoter unmethylated tumor. **C** The violin plot showed the difference of risk score between IDH mutant tumor and IDH wildtype tumor. **D** The top 20 frequently mutated genes in high- and low-risk groups. **E** The differential mutated genes between high- and low-risk groups

We compared the risk scores of GBM for different clinical and molecular subtypes. The results showed that there was no significant difference in the risk score between primary and recurrence GBMs (Fig. 4A), whereas, the score for GBM with IDH mutation and MGMT promoter methylation was significantly lower than that for GBM with wildtype IDH and unmethylated MGMT promoter (Fig. 4B and C). The distribution of the top 20 mutated genes in GBM is illustrated in Fig. 4D. Furthermore, we compared the genes mutations between GBM in the high- and low- risk groups, and the results showed that the mutation frequency of *IDH1*, *SYNE1*, *ADAM29*, *CACNA2D1*, *DICER1*, and *USH2A* in GBM in the low-risk group was significantly higher than that in GBM in the high-risk group (Fig. 4E and F).

**Fig. 5:**
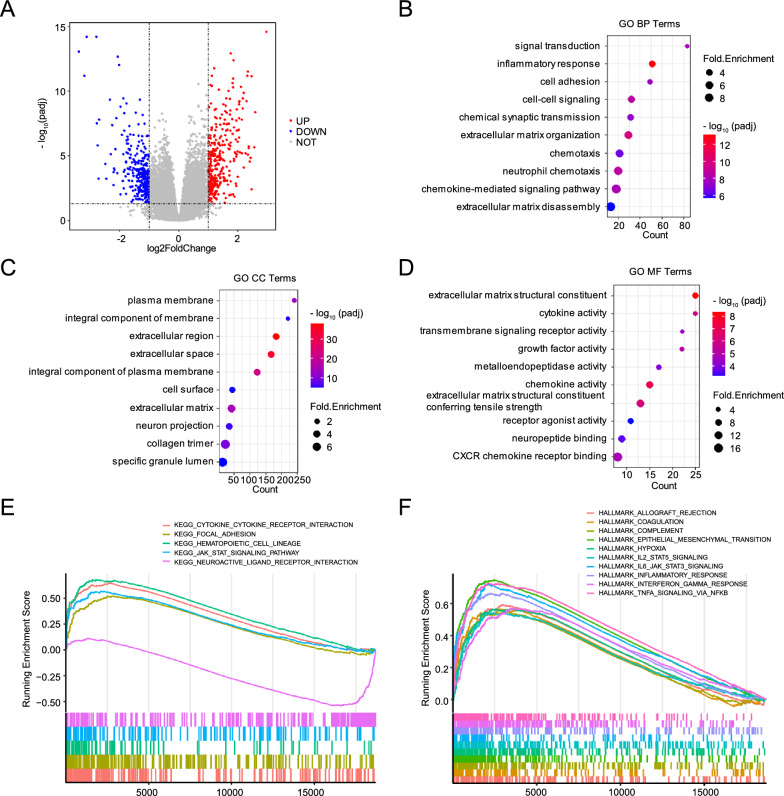
Functional enrichment analysis of our risk score. **A** The volcano plot showed the protein-coding DEGs between high- and low-risk groups based on the TCGA GBM dataset. **B** The top10 biological process terms of GO enrichment analysis of 639 DEGs. **C** The top10 cellular component terms of GO enrichment analysis of 639 DEGs. **D** The top10 molecular function terms of GO enrichment analysis of 639 DEGs. **E** The GSEA enrichment plot based on the KEGG gene sets. **F** The GSEA enrichment plot based on the HALLMARK gene sets

To further understand the underlying mechanism of the risk score, differential expression and functional enrichment analyses were performed. As shown in Fig. 5A, we identified 639 DEGs between the high-risk group and low-risk group, including 328 upregulated and 311 downregulated genes. Based on GO enrichment analysis, we found that these DEGs were significantly enriched in a total of 50 BP terms, 23 CC, and 26 MF terms (adjusted p value < 0.05, Additional file 10: Table S5). The top terms of BP included signal transduction, inflammatory response, chemokine-mediated signaling pathway, and extracellular matrix organization and disassembly (Fig. 5B). For CC, the top terms included plasma membrane, collagen trimer, extracellular region, extracellular space, and extracellular matrix (Fig. 5C). For MF, the top terms included extracellular matrix structural constituents, chemokine activity, cytokine activity, CXCR chemokine receptor binding, metalloendopeptidase activity, and transmembrane signaling receptor activity (Fig. 5D). Additionally, we performed GSEA and found that high-risk patients were not only enriched in pro-cancer-related pathways, such as epithelial mesenchymal transition, hypoxia, and KRAS signaling, but also enriched in immune related pathways, such as inflammatory response, cytokine-cytokine receptor interaction, chemokine signaling pathway and focal adhesion (Fig. 5E and F).

### Correlation between the risk score correlated and immune cell infiltration in GBM

**Fig. 6:**
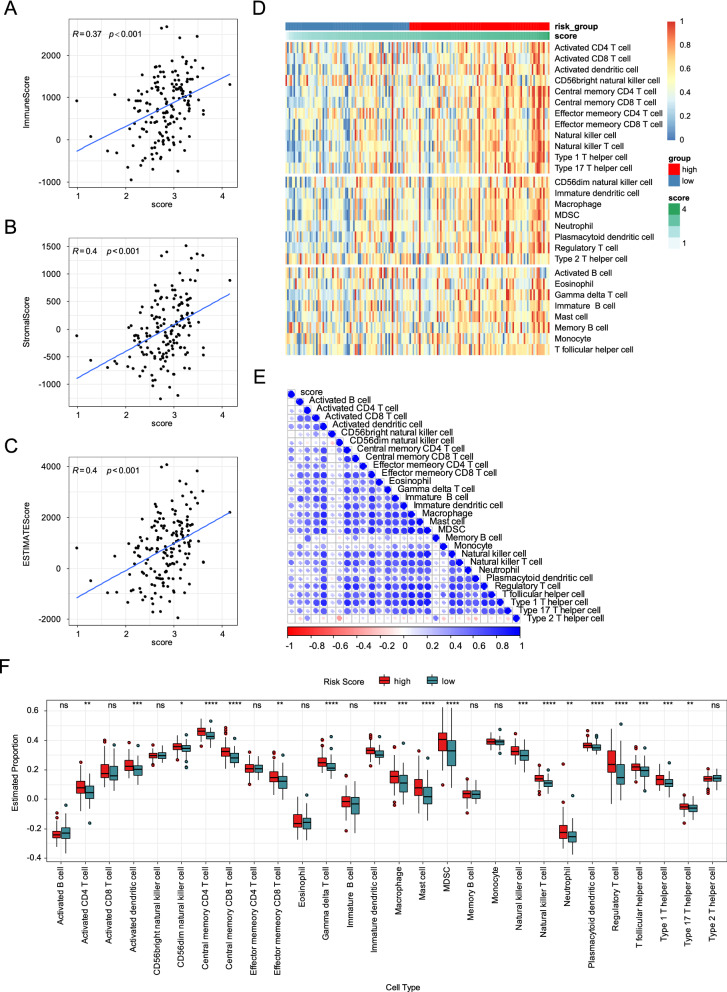
The risk score associated with immune cell infiltration. **A** Scatter plot showed the positive correlation between the risk score and ImmuneScore (Spearman’s rank correlation coefficient). **B** Scatter plot showed the positive correlation between the risk score and StromalScore (Spearman’s rank correlation coefficient). **C** Scatter plot showed the positive correlation between the risk score and ESTAMEScore (Spearman’s rank correlation coefficient). **D** The heatmap plot showed the relationship between risk score and 28 immune cells in the TCGA GBM dataset. **E** The correlations of risk score with abundance of 28 immune cells (Spearman’s rank correlation coefficient). **F** The boxplots showed the relationship between risk score and 28 immune cells in the TCGA GBM cohort

Since functional enrichment analysis showed that the risk score is associated with immune related processes and pathways, we further explored the relationship between risk score and tumor immune microenvironment in GBM. First, we investigated the correlation between risk score and ESTIMATE scores, including ImmuneScore, StromalScroe, and ESTIMATEScore, calculated using the ESTIMATE algorithm in TCGA and GSE16011 GBM cohorts. The results showed that the risk score presented a significantly positive correlation with the ESTIMATED scores in both TCGA and GSE16011 datasets (Fig. 6A–C, Additional file 5: Fig. S5A–C). Furthermore, we estimated the infiltration levels of 28 immune cells in the TCGA and GSE16011 cohorts using the ssGSEA algorithm. Correlation analysis revealed that the risk score positively correlated with most immune cells in both TCGA and GSE16011 datasets (Fig. 6D and E, Additional file 5: Fig. S5D and E). In addition, we found that most of the immune cells were significantly higher in the high-risk group than in the low-risk group based on TCGA cohort (Fig. 6F). Similar results were observed in the GSE16011 cohort (Additional file 5: Fig. S5F).

### Correlation of SSBP1 with GBM cell proliferation and migration

Based on the previous study, we screened out 12 GBM candidates. Indeed, most of these 12 MRGs, including *PLAUR* [21], *RBP1* [22], *MRPL36*, *AGK* [23], *HK1* [23], *APEX1* [24], *NUDT1* [25], and *PHB2* [26], have been reported to play critical roles in the development and invasiveness of GBM. However, the role of SSBP1 in GBM remains unknown. Conversely, multiple studies have demonstrated that SSBP1 is vnvolved in various human cancers and plays an important role [27–30]. Thus, we selected SSBP1 for further experiments.

**Fig. 7:**
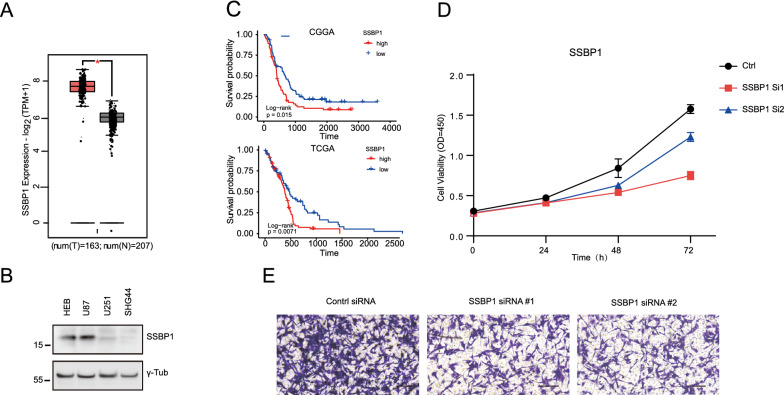
SSBP1 was upregulated and related to proliferation and migration of GBM. **A** Gene expression of SSBP1 between GBM and normal tissue. **B** Expression of SSBP1 in different glioma cell lines detected by western blot analysis. **C** K-M curves of SSBP1 for primary GBM based on the TCGA and CGGA cohorts. **D** Cell proliferation ofU87 cells transferred with control or SSBP1 siRNA was detected by CCK-8 assays. **E** Transwell assay examined cell migration ability of U87 cells transferred with control or SSBP1 siRNA. Scale bar: 200 μm

Bulk RNA analysis also revealed that SSBP1 was significantly upregulated in GBM compared to normal brain tissues based on the GEPIA database (Fig. 7A). WB analysis further confirmed that SSBP1 was only upregulated in U87 cells and not in HEB cells (Fig. 7B). To investigate the relationship between the SSBP1 expression levels and GBM prognosis, the KM survival curves were drawn based on TCGA and CGGA primary GBM databases. The results revealed that SSBP1 was markedly correlated with the OS of patients and a high level of SSBP1 indicated a poor outcome based on TCGA and CGGA GBM dataset (Fig. 7C).

To investigate the biological function of SSBP1 in GBM cells, we knocked down *SSBP1* by transfecting U87 cells with two siRNAs. After transfection with siRNA for 72 h, cell viability was determined using the CCK-8 assay and the results indicated that *SSBP1* silencing significantly inhibited U87 cell proliferation (Fig. 7D). Furthermore, we knocked down *SSBP1* using siRNA in U87 cells and seeded the cells into Transwell chambers. After 48 h, migration assay was performed; compared with negative control, U87 cell migration ability was suppressed on *SSBP1* downregulation (Fig. 7E).

### Effect of SSBP1 on mitochondrial morphology and ROS production

**Fig. 8:**
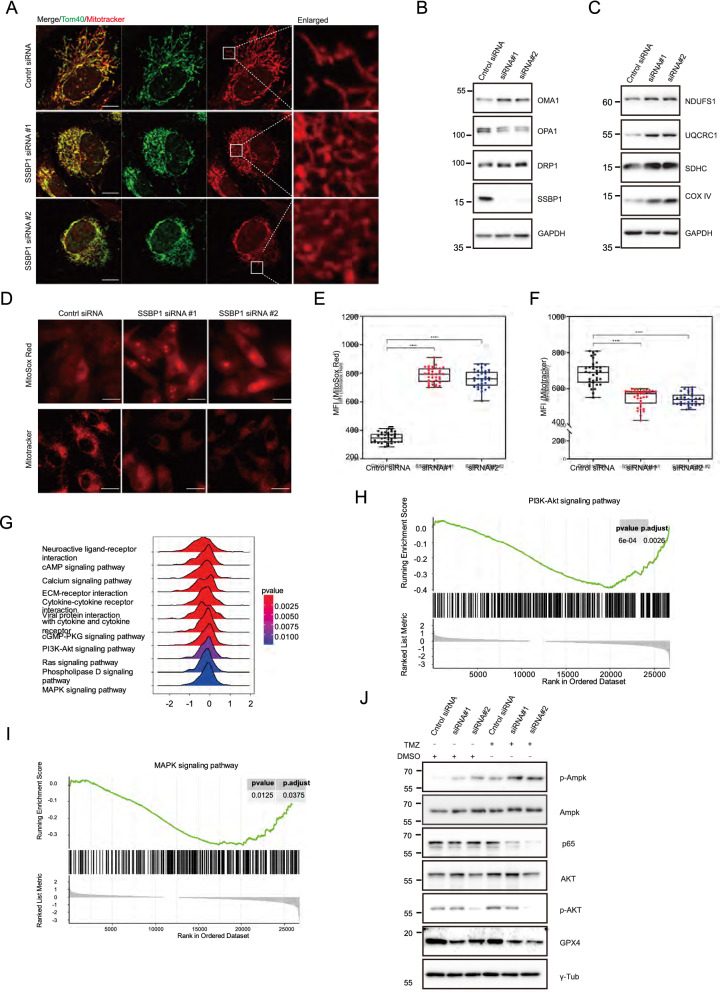
SSBP1 knockdown induced mitochondria dysfunction in U87 cells. **A** Representative confocal image of U87 cells labelled with MitoTracker red (red) and Tom40 (Green). **B** The effects of SSBP1 knockdown on the expressions of mitochondrial morphology mediators. **C** Western blot analysis showing the expression level of NDUFS1, UQCRC1, SDHC and Cox IV. **D** Representive live confocal images of U87 cell transferred with control or SSBP1 siRNA and incubated with MitoTracker Red and MitoSox Red. **E–F** Quantification fluorescence intensity of MitoTracker red and MitoSox. Data are represented as mean ± SD. n = 35. ****P < 0.0001. **G** The enriched KEGG pathways based on the GSEA. **H** The GSEA of PI3K-AKT signalling pathway. **I** The GSEA of MARPK signaling pathway. **J** Western blot analysis showing the expression level of AMPK, p-AMPK and GPX4 in SSBP1 knockdown U87 cells treated with DMSO or TMZ. Scale bars: 10 μm

Since SSBP1 is a prognostic MRG, we next investigated the effect of SSBP1 knockdown on mitochondria in GBM cells. First, we observed that the mitochondria of U87 cells aggregated after transfection with *SSBP1* targeted siRNA for 72 h (Fig. 8A). It is well known that mitochondrial morphology is regulated by central mediator proteins, we determined their expression levels by WB. The results showed that the expressions of OMA1 and OPA1 were upregulated, while that of DRP1 was downregulated after silencing *SSBP1* in U87 cells (Fig. 8B). Furthermore, we determined the expression levels of the five enzyme complexes, constituting the electron transport chain (ETC), and found that the expression levels of NDUFS1 (complex I), UQCRC1 (complex II), SDHC (complex III), and Cox IV (complex IV) were highly upregulated after the knockdown of SSBP1 in U87 cells (Fig. 8C). These results suggested that SSBP1 knockdown upregulated most of the ETC content.

Considering that mitochondrial ROS production results from changes in mitochondrial ETC content or function [31], we speculated whether *SSBP1* knockdown might trigger a notable increase in mitochondrial ROS. Thus, we measured mitochondrial ROS and MMP using live cell imaging, and found that *SSBP1* knockdown resulted in a significant increase in mitochondrial ROS, as well as a dramatic decrease in MMP in U87 cells (Fig. 8D–F.). GSEA analysis also revealed that SSBP1 was enriched in 11 KEGG pathways based on TCGA GBM dataset (Fig. 8G). some of these pathways are associated with ROS, such as the PI3K-Akt (Fig. 8H), MAPK (Fig. 8I), and calcium signaling pathways. Furthermore, we observed AMPK activation and NF-κB pathway downregulation in TMZ-treated *SSBP1* knockdown U87 cells (Fig. 8J). GPX4 expression in TMZ-treated U87 cells was slightly reduced compared with that in DMSO-treated U87 cells. Taken together, these results indicated that *SSBP1* downregulation promotes mitochondrial aggregation and increase ROS production in GBM cells.

### Effect of *SSBP1* downregulation on TMZ sensitivity

**Fig. 9:**
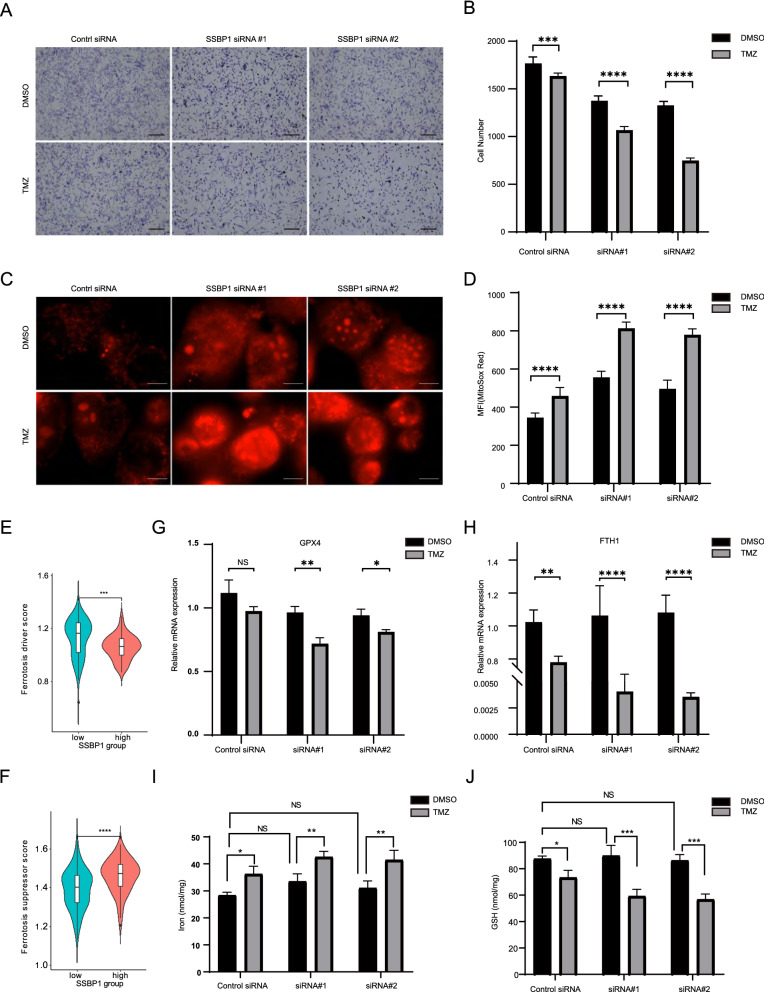
Downregulation of SSBP1 increased TMZ sensitivity and enhanced TMZ induces ferroptosis. **A** Representive images of U87 cells after SSBP1 knockdown, the cells were incubated with TMZ or DMSO and stained with crystal violet. Scale bar: 200 μm. **B** Quantification of the U87 cell number in 8 random sights. Data are represented as mean ± SD. C Representive images of U87 cells after SSBP1 knockdown, the cells were treated with TMZ or DMSO and stained with MitoSox Red. Scale bar: 10 μm. D Quantification fluorescence intensity of MitoSox Red. Data are represented as mean ± SD. n = 30. ****P < 0.0001. **E** The ferroptosis driver score between SSBP1 high group and low group in the CGGA primary GBM samples. **F** The ferroptosis suppressor score between SSBP1 high group and low group in the CGGA primary GBM samples. **G**, **H** Real-time PCR analysis for GPX4 and FTH1 were performed in SSBP1 knockdown U87 cells treated with DMSO or TMZ. The data shown are the mean ± SD from three separate experiments. **I**, **J** Iron content and GSH level of SSBP1 knockdown U87 cells treated with DMSO or TMZ. The data shown are the mean ± SD from three separate experiments. ns = not significant, *P < 0.1, **P < 0.01, ***P < 0.001, ****P < 0.0001

Recent studies have revealed that elevated ROS levels weaken the TMZ resistance in glioma cells [32, 33]. Considering that SSBP1 regulates ROS production, we further explored the effect of SSBP1 knockdown on TMZ sensitivity in GBM cells, and the result showed that *SSBP1* knockdown significantly enhanced TMZ treatment efficacy in U87 cells (Fig. 9A and B). Recently, ferroptosis has become a hot topic and is a form of iron- and ROS-dependent cell death characterized by changes in mitochondrial morphology [34]. Compared with control, the ROS level in SSBP1 knockdown U87 cells increased significantly on TMZ treatment (Fig. 9C and D), indicating the initiation of ferroptosis. We subsequently calculated the ferroptosis score of GBM samples based on the CGGA dataset and found that the ferroptosis driver score of the SSBP1 high group was lower than that of the SSBP1 low group (Fig. 9E), in contrast, the ferroptosis suppressor score of the SSBP1 high group was higher than that of SSBP1 low group (Fig. 9F). Furthermore, the silencing of SSBP1 downregulated the expression of GPX4 and FTH1 at the mRNA level in U87 cells treated with TMZ (Figs. 8J, 9G and H). In addition, *SSBP1* knockdown remarkably increased iron and glutathione level (Fig. 9I and J). These results indicated that SSBP1 knockdown increased the sensitivity of GBM cells to TMZ by enhancing TMZ-induced ferroptosis.

## Discussion

Multiple studies have revealed that MRGs are essential for tumorigenesis and tumor progression and targeting MRGs may be a promising therapeutic approaches for cancer treatment [4]. However, few studies have focused on MRGs and elucidated their role in GBM. In the present study, we constructed and validated a prognostic risk score model for GBM based on 12 DE-MRGs through differential gene expression, univariate Cox, and LASSO-Cox analyse. Our risk model presented excellent performance in predicting the GBM prognosis; the risk score was an independent prognostic factor associated with the clinicopathological and molecular features of GBM. In addition, stratified analysis demonstrated that our risk model could well distinguish the prognosis of patients with radiotherapy or TMZ chemotherapy, which indicated that our model has potential clinical application value and that the 12 DE-MRGs might be involved in chemoradiotherapy resistance in GBM. Indeed, some of these genes have been shown to be related to chemoradiotherapy sensitivity in GBM. Cho et al. reported that the relative expression of APEX1 (APE1) was significantly increased in TMZ-resistant cell lines [35]. Furthermore, functional enrichment and immune cell infiltration analyses revealed that our risk score was significantly associated with inflammatory response, extracellular matrix, pro-cancer-related and immune related pathways, tumor mutation burden and immune cell infiltration in GBM. Therefore, these 12 genes are potential therapeutic targets for GBM treatment.

To the best of our knowledge, our study is the first to identify DE-MRGs in neoplastic and non-neoplastic cells based on single cell data for the first time. Single cell RNA sequencing presents great advantages over bulk RNA sequencing in dissecting heterogeneity in cell populations [36]. Therefore, single cell analysis is more conducive to finding differentially expressed genes among cell types. Based on single cell analysis we screened 201 DE-MRGs in GBM tissues. To further screen out important DE-MRGs in GBM, univariate Cox and LASSO Cox analyses were performed and 12 DE-MRGs were identified, which were used to construct a prognostic risk score model for GBM patients. In addition, our risk score excellently predicted the prognosis of patients with GBM, which in turn indicates that our screening method is feasible. The LASSO is a penalization method used to shrink and select variates for regression [37]. Moreover, our risk model also associated with the prognosis of other cancers and the predictive efficiency of our model was better than that of several reported signatures, including immune-related gene signature, pyroptosis-related gene signature, and autophagy-related gene signature, in GBM. A possible reason behind the difference of predictive power might be due to the involvement of mitochondria in various biological processes, including immune response, hypoxia, and cell death [38]; thus, the screened 12 DE-MRGs are likely to play an important role in GBM and might be therapeutic candidates. Indeed, some of these 12 genes have been reported to be involved in glioma tumorigenesis or to be significant in predicting OS. For example, the expression of RBP1 is significantly elevated in gliomas, and the overexpression of RBP1 enhances the growth, self-renewal ability, invasion, and migration of glioma cells [22]. AGK is markedly overexpressed in glioma and might play an important role in glioma development and progression [23]. LINC00470 inhibits the ubiquitination of HK1, the first key enzyme in the glycolysis pathway, thereby affecting glycolysis, and inhibiting cell autophagy in gliomas [39]. APEX1 (APE1) activity is elevated in gliomas and induces resistance to chemoradiotherapy [35]. *NUDT1*, also known as *MTH1*, is overexpressed in GBM, and its silencing significantly alters glioma cell viability [40]. These studies support the important role of the risk score. Therefore, the members of the 12 DE-MRGs that have not been studied in glioma are worth for further study.

Recently, an increasing number of studies have demonstrated that SSBP1 is significantly correlated with poor patient prognosis and is involved in tumorigenesis, proliferation, and drug sensitivity in certain human cancers [9, 17, 30]. The upregulation of SSBP1 is associated with the aggressiveness of osteosarcoma cells [27]; whereas, its depletion triggers cell death in colorectal cancer cells by affecting the mitochondrial proteome [28]. SSBP1 is a suppressor of triple-negative breast cancer metastasis [29]. SSBP1 participates in mtDNA repair in cancer cells during oxidative stress by interacting with p53 [30]. However, as one of the 12 DE-MRGs, the role of SSBP1 in GBM remains unclear. Therefore, we investigeted the role of SSBP1 in GBM. Based on bioinformatic analysis, we found that SSBP1 is aberrantly upregulated in GBM tissue and significantly related to the poor prognosis of primary GBM patients. Previous studies have shown that SSBP1 is essential for mtDNA maintenance and replication. mtDNA can lead to devastating, heritable, and multisystem diseases that have different tissue-specific presentations and are important in the initiation and maintenance of tumorigenesis in GBM [41–43]. Therefore, we focused on exploring the role of SSBP1 in mitochondrial function. Our study demonstrates that silencing SSBP1 expression inhibits GBM cell proliferation and migration. SSBP1 enhances mtDNA replication under physiological conditions, resulting in ATP generation through oxidative phosphorylation [42]. Therefore, we speculated that SSBP1 knockdown might affect mitochondrial ROS production by regulating oxidative phosphorylation.

In recent years, many biomaterials with new technologies have been developed for cancer treatment and have shown promising application prospects. Nanotechnology-based approaches exhibit higher efficacy, higher target specificity, and great potential to bypass the limitations of traditional therapies [44]. As a factory of energy involved in the proliferation of cancer cells, mitochondria are naturally regarded as an important target for cancer therapeutics. Th mitochondria in cancer cells are characterized by ROS overproduction, which promotes cancer development. Recently, multiple novel agents specific for ROS targets have been shown to efficiently maximize chemotherapy efficacy and minimize side effects [45, 46]. ROS-responsive micro- and nano-particles specifically release their drug cargo guided by ROS concentration, which is enhanced in the cellular environment within specific tumors, and thus, show marked cytotoxicity for cancer cells compared to non-ROS responsive molecules [14]. Hence, a better understanding of the role of mitochondrial ROS in GBM will help identify novel therapeutic targets. In this study, we demonstrated that *SSBP1* knockdown promotes ROS production and alters MMP in GBM cells, which is consistent with the results of *SSBP1* knockdown in other cancers [28–30]. Ubiquinol-cytochrome c reductase hinge protein (UQCRH) regulates electron transfer from cytochrome c1 to cytochrome c, and its upregulation enhances ROS production [47]. UQCRC1 is a subunit of UQCRH, and its upregulation can result in enhanced ROS production. ROS production may also be elevated due to the upregulation of mitochondrial ETC function, as implied by upregulation of other ETC components. Our results indicated that SSBP1 might regulates ROS by regulating the expression of UQCRC1, however, the exact mechanisms underlying these processes require further study.

We found that mitochondria of U87 cells with SSBP1 knockdown were fragmented and aggregated. These changes seem to correlate with the expression of mitochondrial morphology mediators, such as the upregulation of OMA1 and DRP1 and downregulation of OPA1. Since we demonstrated that SSBP1 is a potential mitochondrial biomarker of GBM, we further investigated whether it could also be a therapeutic target for GBM. Although TMZ is the first-line chemotherapy for GBM, its efficacy is limited by acquired chemoresistance. Oliva et al. found that TMZ-dependent acquired chemoresistance might be due to a mitochondrial adaptive response to TMZ genotoxic stress with a major contribution from cytochrome c oxidase [48]. Lomeli et al. have also reported that TMZ can lead to mitochondrial dysfunction, oxidative stress, and apoptosis [49]. A recent study demonstrated that TMZ can suppress tumor cell proliferation by inducing ferroptosis, which might be a result of ROS accumulation [50]. Thus, targeting mitochondrial ROS may overcome the TMZ resistance and improve TMZ efficacy. Recently, ferroptosis has become a hot research topic, and several studies have investigated ferroptosis-related biomarkers through bioinformatics analysis or experiments [51, 52]. Here, we combined bioinformatics and experimental methods to confirm that *SSBP1* knockdown enhances ROS production to trigger ferroptosis in U87 cells. Furthermore, upon TMZ treatment, *SSBP1* knockdown activated the AMPK pathway and inhibited the NF-κB pathway in U87 cells. AMPK pathway activation and NF-κB pathway inhibition have been reported to enhance the anti-cancer effects of chemotherapy [53, 54]. Therefore, we can conclude that *SSBP1* knockdown increases TMZ sensitivity by promoting mitochondrial ROS to trigger ferroptosis and regulate the AMPK and NF-κB pathways. This result implied that the strategy of combining an SSBP1 inhibitor with TMZ would benefit tumour treatment by enhancing TMZ sensitivity, however, additional efforts are required to translate this strategy into the clinical setting to benefit GBM patients. Further studies on the mechanisms of MRGs and ferroptosis in TMZ resistance would provide new ideas for the clinical reversal of TMZ resistance and improve the efficacy of chemotherapy.

## Conclusions

We constructed a prognostic model based on 12DE-MRGS that can predict the prognosis of GBM with excellent performance. We also determined the molecular and immunological characteristics of our prognostic model, thus providing potential therapeutic targets for GBM treatment. Furthermore, we demonstrated that the downregulation of *SSBP1* in GBM suppresses tumor proliferation and results in mitochondrial dysfunction. Therefore, our results suggest that SSBP1 as a potential therapeutic target for GBM by enhancing TMZ sensitivity through ROS-mediated ferroptosis.

## Supplementary Information

**Additional file 1: Fig. S1.** Validation of DE-MRGs related prognostic risk model for GBM. A, E Time dependent ROC curves for 12 DE-MRGS prognostic model in the GSE147352 and GSE16011 GBM cohort. B, F The distribution of risk scores, survival time, and status of GBM patients in the GSE147352 and GSE16011 GBM cohort. C, GThe heatmap of the 12 model DE-MRGs in the GSE147352 and GSE16011 GBM cohort. D, H Kaplan–Meier curves for OS in the GSE147352 and GSE16011 GBM cohort stratified by 12 DE-MRGs model in high- and low-risk.

**Additional file 2: Fig. S2.** The predictivity of 12 DE-MRGs model for other cancers. A The univariate cox analysis showed that the risk score, based on our 12 DE-MRGs, significantly associated with other 12 types of TCGA cancers. B Kaplan–Meier curves for OS in the other 12 types of TCGA cancers. The patients were divided in to high- and low-risk groups based on the median value of risk score.

**Additional file 3: Fig. S3.** The time dependent ROC curves for other three prognostic models. A Time dependent ROC curves for immune-related gene signature in the TCGA GBM cohort. B Time dependent ROC curves for pyroptosis-related gene signature in the TCGA GBM cohort. C Time dependent ROC curves for autophagy-related gene signature in the TCGA GBM cohort.

**Additional file 4: Fig. S4.** Validation of independence of our risk score as a prognostic factor. A The forest plot showed the univariate cox analysis using risk score, age, radiotherapy, TMZ chemotherapy, and gender as variates in the GSE16011 GBM cohort. B The forest plot showed the multivariate cox analysis using risk score, age, radiotherapy, and TMZ chemotherapy as variates in the GSE16011 GBM cohort. C Stratified OS analysis in GSE16011 GBM patients with different age based on our risk model. D Stratified OS analysis in GSE16011 GBM patients with radiotherapy or not based on our risk model. E Stratified OS analysis in TCGA GBM patients with TMZ chemotherapy or not based on our risk model.

**Additional file 5: Fig. S5.** Validation of the association between risk score and immune cell infiltration. A Scatter plot showed the positive correlation between the risk score and ImmuneScore (Spearman’s rank correlation coefficient) in the GSE16011 GBM cohort. B Scatter plot showed the positive correlation between the risk score and StromalScore (Spearman’s rank correlation coefficient) in the GSE16011 GBM cohort. C Scatter plot showed the positive correlation between the risk score and ESTAMEScore (Spearman’s rank correlation coefficient) in the GSE16011 GBM cohort. D The heatmap plot showed the relationship between risk score and 28 immune cells in the GSE16011 GBM dataset. E The correlations of risk score with abundance of 28 immune cells (Spearman’s rank correlation coefficient) in the GSE16011 GBM dataset. F The boxplots showed the relationship between risk score and 28 immune cells in the GSE16011 GBM cohort.

**Additional file 6: Table S1.** The mitochondria-related genes extracted from the uniprot database.

**Additional file 7: Table S2.** The primer sequences used in the study.

**Additional file 8: Table S3.** The 201 DE-MRGs between neoplatstic and non-neoplastic cells.

**Additional file 9: Table S4.** The 21 prognositc DE-MRGs based on the TCGA GBM cohort.

**Additional file 10: Table S5.** GO enrichment analysis by using DAVID.

## Data Availability

The datasets used and/or analysed during the current study are available from the corresponding author on reasonable request.

## Abbreviations

GBM: Glioblastoma
DRP1: Dynamin-related protein 1
GSEA: Gene set enrichment analysis
HR: Hazard ratio
MGMT: O6-methylguanine-DNA-methyltransferase
OS: Overall survival
SSBP: Single strand binding protein
siRNA: Small interference RNA
mtDNA: Mitochondrial DNA
MMP: Mitochondrial membrane potential
SSBP1: Single-stranded DNA-binding protein 1
ROS: Reactive oxygen species
TMZ: Temozolomide
ETC: Electron transport chain
MRG: Mitochondria-related gene
OXPHOS: Oxidative phosphorylation
GO: Gene ontology
UQCRH: Ubiquinol-cytochrome c reductase hinge protein
LASSO: Least absolute shrinkage and selection operator
TCGA: The cancer genome atlas
CGGA: Chinese glioma genome atlas
GEO: Gene expression omnibus
