# Supplementary figures and images for: Identification of SSBP1 as a ferroptosis-related biomarker of glioblastoma based on a novel mitochondria-related gene risk model and in vitro experiments
Source: J Transl Med. 2022 Sep 30;20:440. doi: 10.1186/s12967-022-03657-4 (PMC9524046; doi:10.1186/s12967-022-03657-4)

A

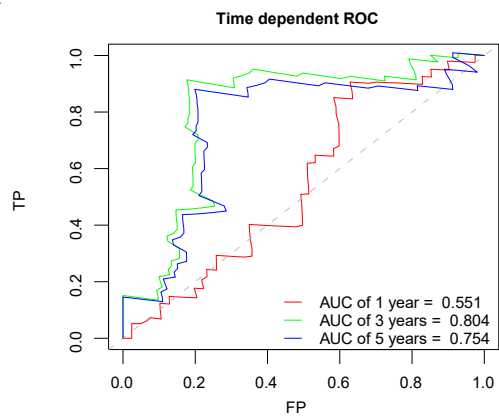

B

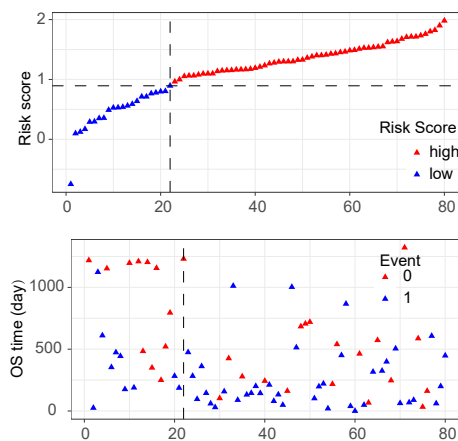

C

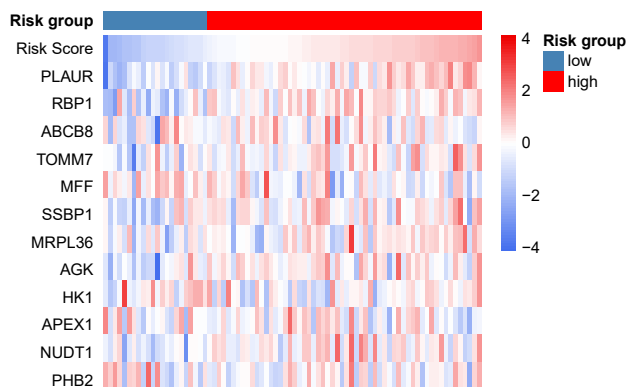

D

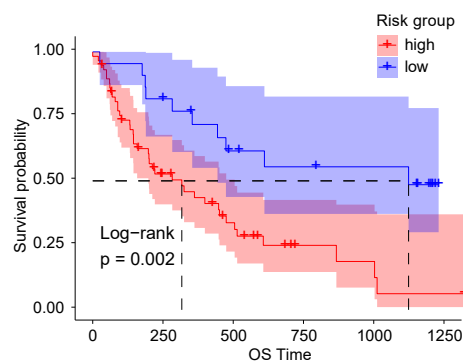

E

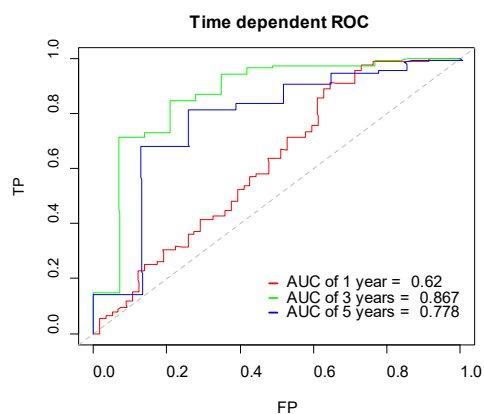

F

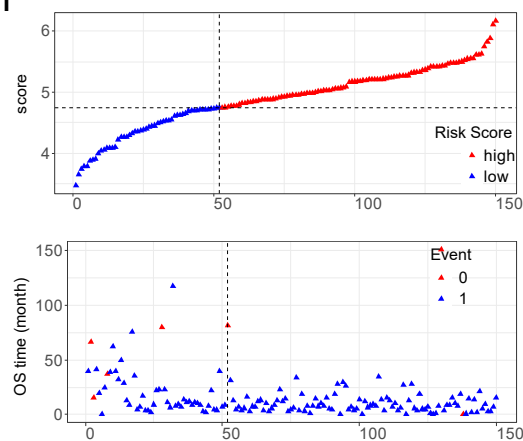

G

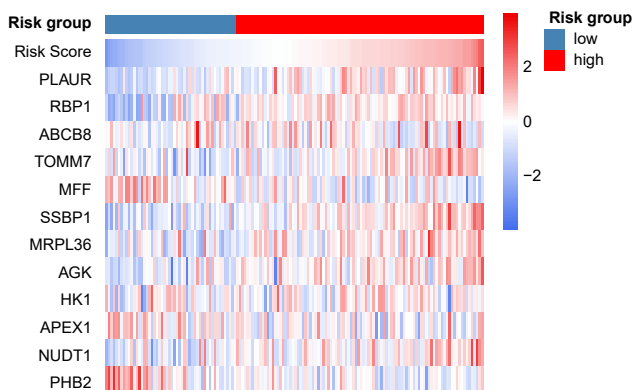

H

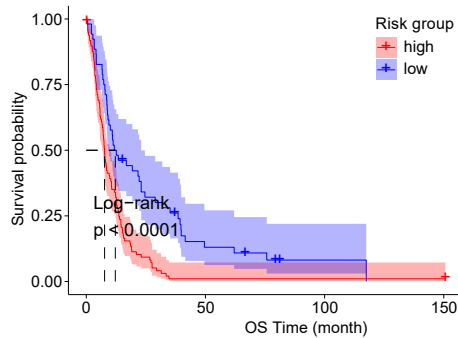

Supplement: Supplementary file 1 — Additional file 1: Fig. S1. Validation of DE-MRGs related prognostic risk model for GBM. A, E Time dependent ROC curves for 12 DE-MRGS prognostic model in the GSE147352 and GSE16011 GBM cohort. B, F The distribution of risk scores, survival time, and status of GBM patients in the GSE147352 and GSE16011 GBM cohort. C, GThe heatmap of the 12 model DE-MRGs in the GSE147352 and GSE16011 GBM cohort. D, H Kaplan–Meier curves for OS in the GSE147352 and GSE16011 GBM cohort stratified by 12 DE-MRGs model in high- and low-risk. [file 12967_2022_3657_MOESM1_ESM.pdf]

A

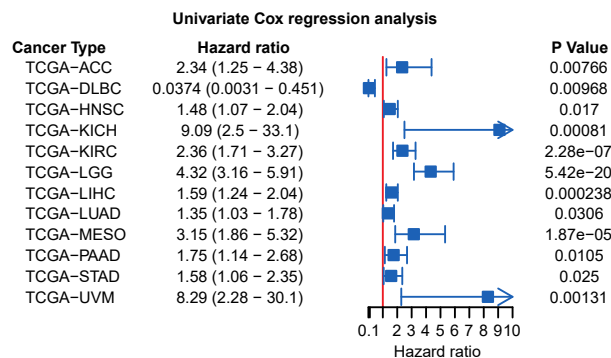

B

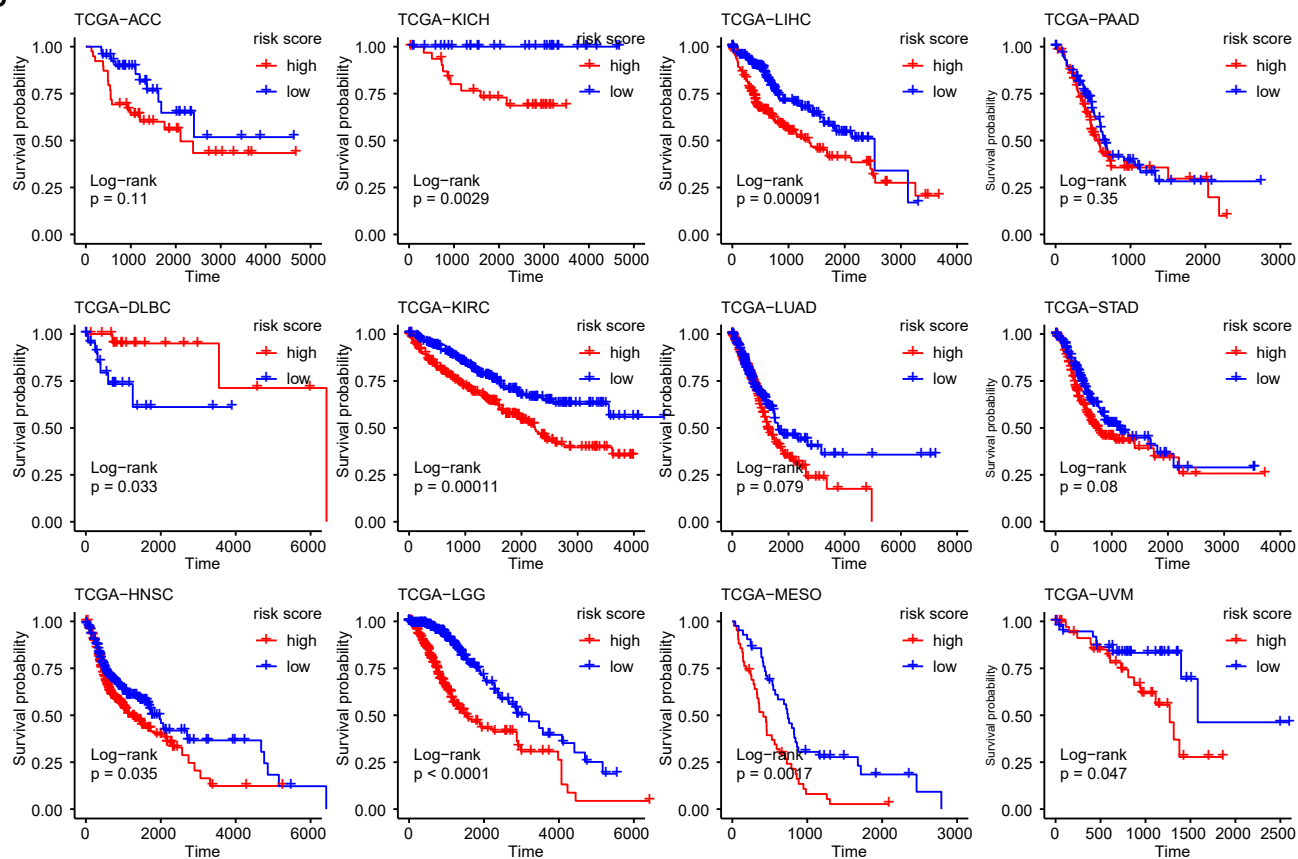

Supplement: Supplementary file 2 — Additional file 2: Fig. S2. The predictivity of 12 DE-MRGs model for other cancers. A The univariate cox analysis showed that the risk score, based on our 12 DE-MRGs, significantly associated with other 12 types of TCGA cancers. B Kaplan–Meier curves for OS in the other 12 types of TCGA cancers. The patients were divided in to high- and low-risk groups based on the median value of risk score. [file 12967_2022_3657_MOESM2_ESM.pdf]

A

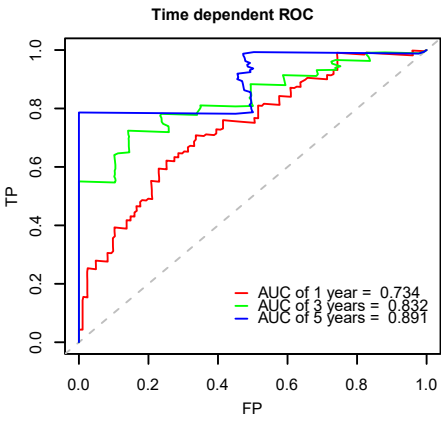

B

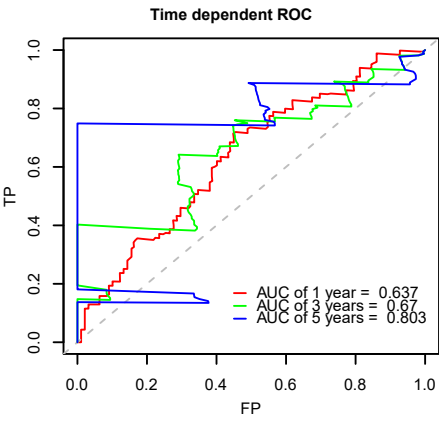

C

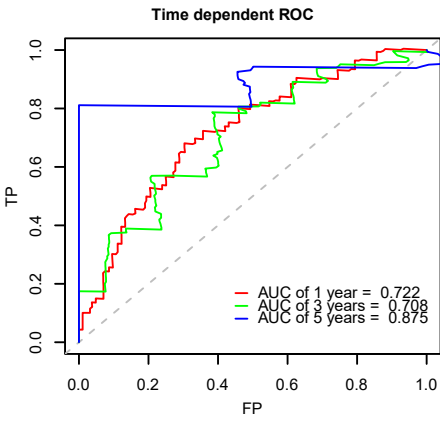

Supplement: Supplementary file 3 — Additional file 3: Fig. S3. The time dependent ROC curves for other three prognostic models. A Time dependent ROC curves for immune-related gene signature in the TCGA GBM cohort. B Time dependent ROC curves for pyroptosis-related gene signature in the TCGA GBM cohort. C Time dependent ROC curves for autophagy-related gene signature in the TCGA GBM cohort. [file 12967_2022_3657_MOESM3_ESM.pdf]

A

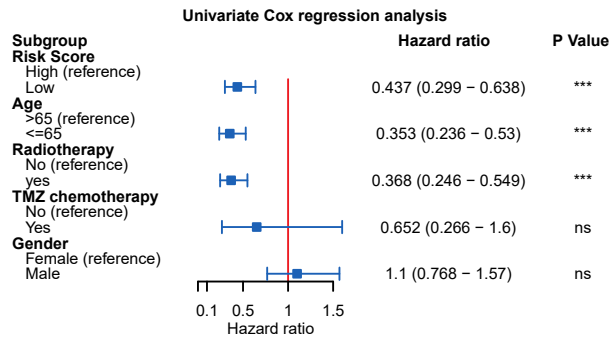

B

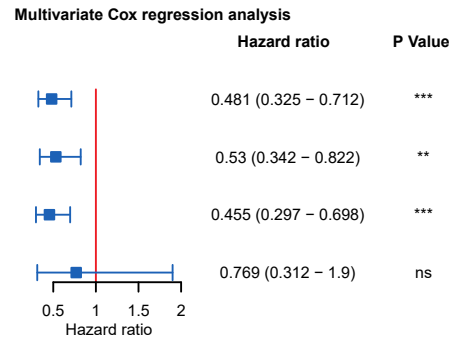

C

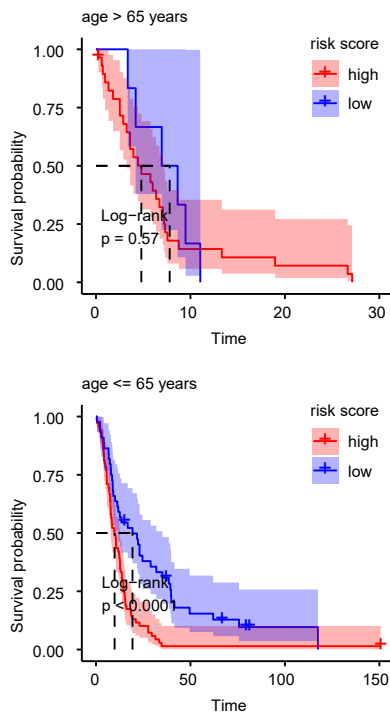

D

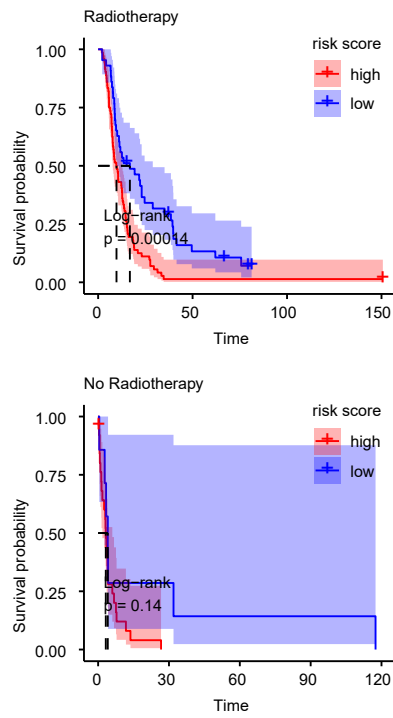

E

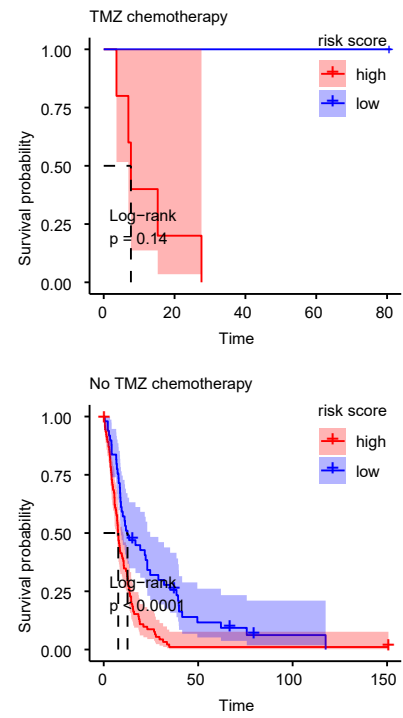

Supplement: Supplementary file 4 — Additional file 4: Fig. S4. Validation of independence of our risk score as a prognostic factor. A The forest plot showed the univariate cox analysis using risk score, age, radiotherapy, TMZ chemotherapy, and gender as variates in the GSE16011 GBM cohort. B The forest plot showed the multivariate cox analysis using risk score, age, radiotherapy, and TMZ chemotherapy as variates in the GSE16011 GBM cohort. C Stratified OS analysis in GSE16011 GBM patients with different age based on our risk model. D Stratified OS analysis in GSE16011 GBM patients with radiotherapy or not based on our risk model. E Stratified OS analysis in TCGA GBM patients with TMZ chemotherapy or not based on our risk model. [file 12967_2022_3657_MOESM4_ESM.pdf]

A

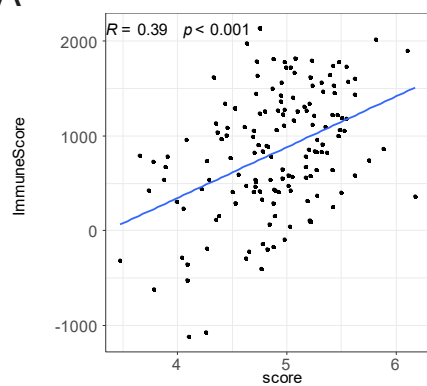

B

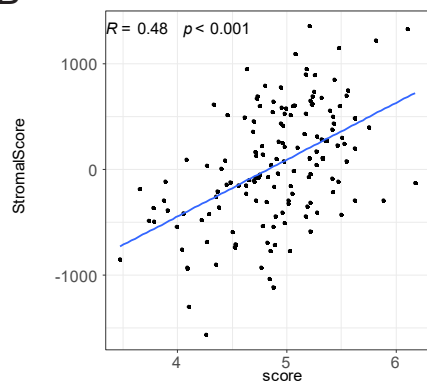

C

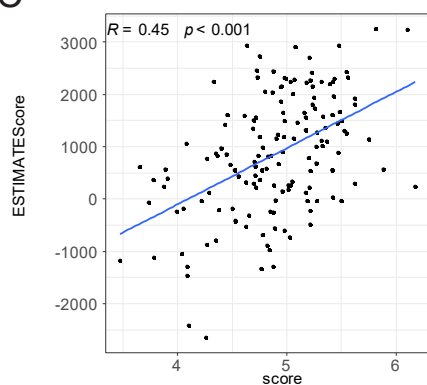

F

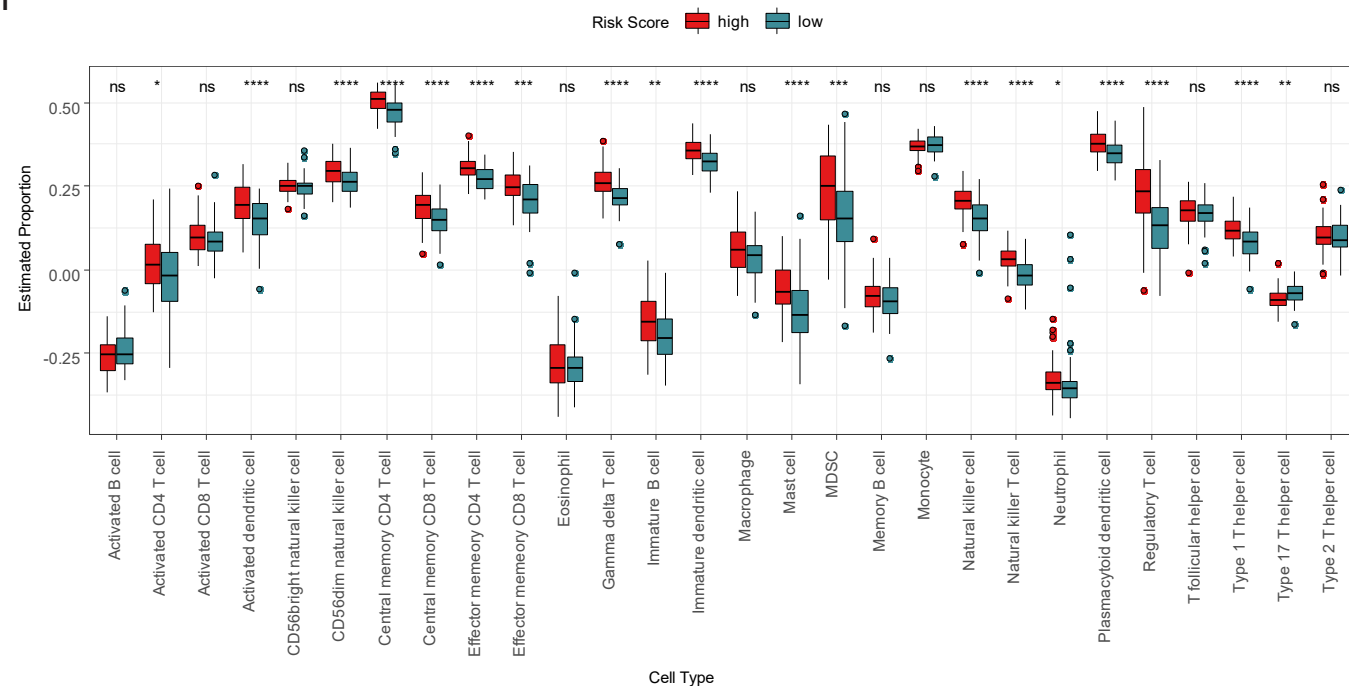

D

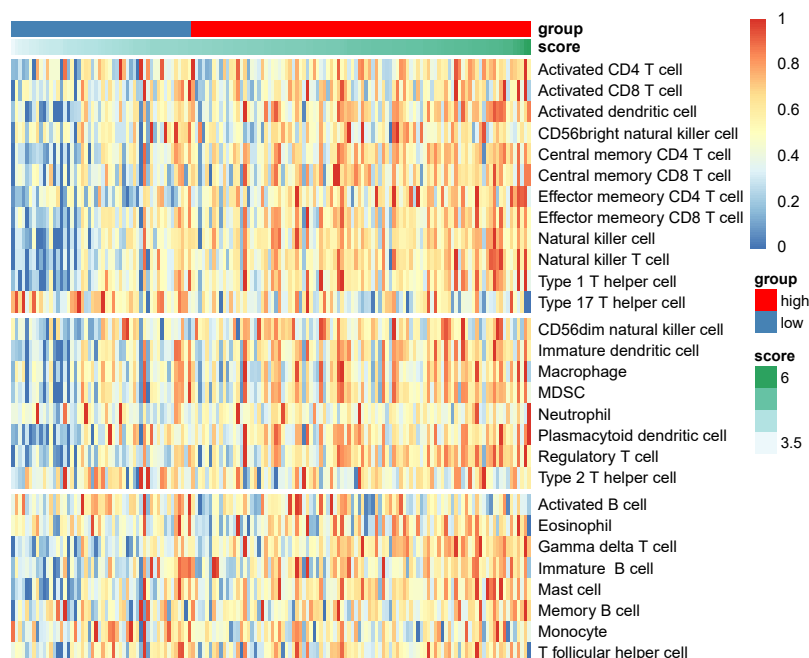

E

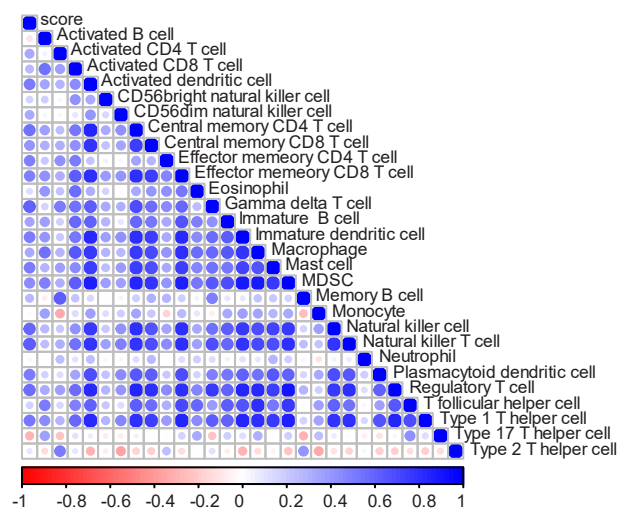

Supplement: Supplementary file 5 — Additional file 5: Fig. S5. Validation of the association between risk score and immune cell infiltration. A Scatter plot showed the positive correlation between the risk score and ImmuneScore (Spearman’s rank correlation coefficient) in the GSE16011 GBM cohort. B Scatter plot showed the positive correlation between the risk score and StromalScore (Spearman’s rank correlation coefficient) in the GSE16011 GBM cohort. C Scatter plot showed the positive correlation between the risk score and ESTAMEScore (Spearman’s rank correlation coefficient) in the GSE16011 GBM cohort. D The heatmap plot showed the relationship between risk score and 28 immune cells in the GSE16011 GBM dataset. E The correlations of risk score with abundance of 28 immune cells (Spearman’s rank correlation coefficient) in the GSE16011 GBM dataset. F The boxplots showed the relationship between risk score and 28 immune cells in the GSE16011 GBM cohort. [file 12967_2022_3657_MOESM5_ESM.pdf]
