# Supplementary material for: Identification of SSBP1 as a ferroptosis-related biomarker of glioblastoma based on a novel mitochondria-related gene risk model and in vitro experiments
Source: J Transl Med. 2022 Sep 30;20:440. doi: 10.1186/s12967-022-03657-4 (PMC9524046; doi:10.1186/s12967-022-03657-4)
Supplement: Supplementary file 7 — Additional file 7: Table S2. The primer sequences used in the study. [file 12967_2022_3657_MOESM7_ESM.docx]

Supplementary table 2. The primer sequences used in the study.

| Gene | Forward primer | Reverse primer |
| --- | --- | --- |
| SSBP1 | ACTGGGTGATGTCAGTCAAAAG | TGCTTGTCGCCTCACATTATT |
| GPX4 | ACAAGAACGGCTGCGTGGTGAA | GCCACACACTTGTGGAGCTAGA |
| FTH1 | CTTCCATGCTGAAGCCACGCTT | GCACACTCCATTGCATTCAGCC |
| β-Actin | ACCAACTGGGACGACATGGAG | GTGGTGGTGAAGCTGTAGCC |
